# Enhancing Anti-cancer Activity: Green Synthesis and Cytotoxicity Evaluation of Turmeric-Gold Nanocapsules on A549 Lung Cancer Cells

**DOI:** 10.7759/cureus.43087

**Published:** 2023-08-07

**Authors:** Saikat Dutta, Saumit Kumar Mitra, Aritri Bir, Prabha T R, Arindam Ghosh

**Affiliations:** 1 Microbiology, Vels Institute of Science, Technology & Advanced Studies, Chennai, IND; 2 Biochemistry, Indian Institute of Technology Kharagpur, Kharagpur, IND; 3 Biotechnology, Indian Institute of Technology Madras, Chennai, IND

**Keywords:** nanomedine in cancer, lung cancer cell line, a549 cell line, green synthesis, anticancer drugs, nanotechnology in lung cancer, ethylcellulose, gold nanocapsule, gold nanoparticle, turmeric

## Abstract

Background

Lung cancer remains a major global health concern, with a notable increase in new cases in recent years. This study aims to investigate the cytotoxic effects of polymeric turmeric-gold nanocapsules on A549 human lung cancer cells, utilizing green-synthesized gold nanoparticles from *Curcuma longa L*. and ethyl cellulose-based nanocapsules.

Methods

Gold nanoparticles were synthesized using the aqueous root extract of *Curcuma longa L*., and the resulting nanoparticles were characterized using UV-Vis, fourier transform infrared spectroscopy (FTIR), transmission electron microscopy (TEM), and energy dispersive x-ray (EDX) techniques. Subsequently, polymeric nanocapsules of turmeric with encapsulated gold nanoparticles were prepared. The cytotoxicity of these nanocapsules was evaluated using the 3-(4,5-dimethylthiazol-2-yl)-2,5-diphenyl-2H-tetrazolium bromide (MTT) assay on both A549 lung cancer cell lines and normal cell lines.

Results

The turmeric-gold nanocapsules exhibited a half maximal inhibitory concentration (IC50) value of 40 μg/ml, while the gold nanoparticles alone showed an IC50 value of 60 μg/ml when tested on A549 cells. Furthermore, apoptosis was observed in A549 cells treated with turmeric-gold nanocapsules. The combination of gold nanoparticles and turmeric polymer (gold turmeric nanocapsules) demonstrated a more potent anti-cancer effect on the lung cancer cell line, with an IC50 value of 40 μg/ml compared to green-synthesized gold nanoparticles (IC50 of 60 μg/ml).

Conclusion

The utilization of polymeric nanocapsules of turmeric, with green-synthesized gold nanoparticles, presents a promising solution to overcome the limited water solubility of turmeric. The results suggest that the combination of gold nanoparticles and turmeric enhances the cytotoxic effects on A549 human lung cancer cells. These findings contribute to the potential application of turmeric-gold nanocapsules as a novel therapeutic approach in lung cancer research.

## Introduction

Lung malignancy is the most common cancer in the world after breast cancer. Abrupt variations in way of life, natural contamination, and smoking have been firmly identified as the cause of lung malignant growth [1]. Handling lung malignant growth is normally driven by phase, albeit singular components, for example, general well-being and coinciding ailments, are likewise significant. Chemotherapy is advantageous in most phases of the malady, although it and radiation treatment are curative in just a handful of patients. Even though these treatments are highly fruitful still they come up with serious side effects as a by-product [2]. Medicinal plants find themselves a great source of a good number of bio-actives that act as excellent agents against cancer as they have the adeptness to synchronize the molecular mechanisms and various signaling pathways involved in carcinogenesis such as oxidation, inflammation, apoptosis, cell proliferation, cell cycle, invasion, metastasis and angiogenesis [3]. This problem has been resolved with the arrival of nanotechnology, which has made a noted impression on the development of novel drug delivery systems [4].

Curcumin (turmeric) is a polyphenol procured from the most part of turmeric, the rhizome of *Curcuma longa L*, which is generally utilized in culinary as a flavor and shading added substance because of its trademark taste and profound yellow coloration [5]. Due to several medicinal properties like antioxidant and anti-inflammatory activity, turmeric is utilized in pharmacy sectors [6]. And also curcumins had shown an assortment of natural properties, for example, cancer prevention agents, antimicrobial, and antitumor properties. Nanoparticle-primarily based targeted drug transport method has the ability for rendering curcumin in particular at the usage of an outside magnetic field [7,8]. It can also enhance availability and keep away from the pitfalls of terrible solubility [9,10]. Gold nanoparticles have excellent antibacterial & anticancer activity [6]. The green gold nanoparticles from *C. longa* show less cytotoxicity and helps in cancer study [11]. To preserve and improve human health, nanomedicine has got a distinct and significant place as it uses molecular tools and molecular knowledge of the very human physique [12]. Nanoparticles are developed aiming at improvising the pharmacokinetics of modern drugs by refining their competence, solidity, and solubility, reducing their toxicity, besides the target site specificity [13]. It is a piece of science wherein we can make 100 nm held onto particles and it gives generally changed different materials. Novel metal nanoparticles like gold nanoparticles (AuNPs) are an assuring response to malignant therapy [14]. Gold nanoparticles hold an area of attention considering the exceptional properties (optical-based morphology, electrical, and alluring ability) that can be linked with anticancer therapies, materials for biosensors, composite fibers, cryogenic conducting materials, and electronic fragments [15]. In ongoing decennary, polymer-based nanocapsules have been read and implemented for fundamental and also topical medication conveyance. The primary scholarly investigations announcing the arrangement of nanocapsules go back to the 1980s. Nanocapsules are frameworks in which the medication is limited to a depression encompassed by a one-of-a-kind nontoxic polymer layer. When polymer-based nanoparticles contain a polymeric divider made out of non-ionic surfactants, macromolecules are named nanocapsules [16]. Nanocapsules consist of a polymer membrane and an oil core containing drugs that can diffuse in response to environmental, chemical, thermal, or biological triggers under appropriate conditions [17]. Nonetheless, seen from a general level, they can be characterized as a nano-vesicular framework that displays a regular core-shell structure in which the medication is kept to supply or inside a core encompassed by a polymer film or covering and suitable for quality drug delivery of hydrophobic drug like curcumin. The core can contain the dynamic substance in a fluid structure or as a sub-atomic scattering. In like manner, this repository can be lipophilic and hydrophobic as indicated by the readiness strategy and crude materials utilized. Biodegradable polymer ethyl cellulose comes as one inside the list of the supreme helpful polymer utilize for tranquilizing conveyance purposes [18]. Ethylcellulose shares cohabitation with an extremely modest sum of water-indissoluble polymers that are affirmed for global pharmaceutical usages and is most as often as possible utilized in broadened discharge strong dose definitions. Notwithstanding being helpful in an assortment of pharmaceutical applications, ethylcellulose likewise gives definition adaptability by obliging a scope of sub-atomic loads and can be mixed for the middle of road viscosities. The nearness of oil in the nanocapsules prompts a vesicular structure which gives a matricidal association of the polymeric chains [19]. In light of the above foundation, the expansive point of this theory was to investigate the capability of nanocapsules in the conveyance of herbals or their bioactive in lung malignant growth cells.

## Materials and methods

Materials

The following materials were obtained from Sigma-Aldrich: Dulbecco's modified eagle medium (DMEM), fetal bovine serum (FBS), 0.25% trypsin-ethylenediamine tetraacetic acid (EDTA), streptomycin, penicillin, dimethyl sulfoxide (DMSO), 3-(4,5-dimethylthiazol-2-yl)-2,5-diphenyltetrazolium bromide) (MTT), phosphate buffer, acridine orange, ethidium bromide (AO/EB), Chloroauric acid/gold chlorides (HAuCl_4_), ethylcellulose (EC), dichloromethane (CH_2_CL_2_), and polyvinyl alcohol (PVA). A549 lung cancer cell lines were obtained from the National Center for Cell Sciences (NCCS), Pune, Maharashtra, India.

Biological synthesis of gold nanoparticles and gold nanocapsules

*Curcuma longa* roots were obtained from the market, washed with double-distilled water, dried in the shade, and ground to a fine powder. The powder (2 g) was mixed with 30 mL of sterilized distilled water and placed in a water bath for 45 minutes. The extract was then filtered using Whatman filter paper. A solution of HAuCl4 (1 mM, 9 mL) was gently mixed with the filtrate and incubated overnight at room temperature in the dark. Turmeric gold nanocapsules were synthesized using the solvent evaporation method [20]. The polymer solution was emulsified into a liquid phase in the first step, followed by the evaporation of the polymer solvent, resulting in the precipitation of the polymer as nanocapsules. For the EC/turmeric-Au investigation, different concentrations (20, 40, 60, 80, and 100 µg/mL) of turmeric were added to 1 mL of the nanogold solution, and the mixture was then added to the ethyl cellulose dichloromethane solution (300 mg ethyl cellulose + 5 mL dichloromethane) along with 1% polyvinyl alcohol for emulsification. The solvent was evaporated until it turned clear. The synthesized gold nanocapsules were collected after filtration, washed with demineralized water to remove any impurities, and stored overnight at 40°C. The nanocapsules were stored in desiccators at 25°C.

Characterization of nanoparticles and nanocapsules

The formation and stability of gold nanoparticles (AuNPs) were confirmed using UV-Visible spectroscopy. The absorbance spectra of the synthesized AuNPs and the aqueous extract of *C. longa* roots mixed with the chloroauric acid solution were recorded in the range of 300 to 700 nm using a Hitachi U-2900 spectrophotometer. Transmission electron microscopy (TEM) was performed by drop-casting the gold nanoparticle suspension onto a carbon-coated copper TEM grid (Ted Pella). The grid was air-dried at ambient conditions for two minutes and stored in a desiccator. The size distributions of the nanoparticles were determined using image analysis with the ImageJ software package. TEM analysis was conducted using a Hitachi 4800 transmission electron microscope. The presence of various elements and the elemental composition of the gold nanoparticles (AuNPs) were determined using energy dispersive x-ray (EDX) analysis. The synthesized AuNPs were centrifuged, and the supernatant was discarded. The pellet was collected, incubated at 50°C to remove excess water, and allowed to settle at ambient temperature. X-ray diffraction analysis was performed using a SEIFERT JSO-DEBYE FLEX 2002 powder x-ray diffractometer to study the crystalline form of the gold nanoparticles (AuNPs). Lyophilized gold nanoparticles were analyzed using x-ray diffractometry in the 2θ range of 30° to 70° with a step size of 0.04° per second. Fourier transform infrared spectroscopy (FTIR) was conducted to identify the biomolecules present in the gold nanoparticle sample derived from *Curcuma longa*. The prepared lyophilized gold nanoparticles were analyzed using FTIR spectroscopy (Hitachi 270-50) in the range of 400 to 4000 cm−1 using KBr pellets. The free radical scavenging activity of the synthesized gold nanoparticles was assessed using the 1,1-diphenyl-2-picrylhydrazyl (DPPH) assay [21]. Different concentrations of the gold nanoparticles were mixed with a DPPH solution (0.002% in methanol) in separate tubes. After incubation in the dark at room temperature for 30 minutes, the absorbance was measured at 517 nm using a UV-Vis spectrophotometer. The scavenging activity was calculated using the formula: Scavenging Activity (%) = [(A − B)] × 100/A, where A is the absorbance of the DPPH control and B is the absorbance of DPPH in the presence of the extract/standard. Transmission electron microscopy was used to detect the prepared nanocapsules (EC-Au) morphology using TEM analysis (Hitachi 4800 TEM). The gold nanocapsules were characterized using FTIR. The presence of specific functional groups in the nanocapsules was confirmed by FTIR analysis.

Cell line based experiments

The A549 cells were cultured in DMEM supplemented with 10% heat-inactivated FBS, streptomycin, penicillin-G (100 µg/mL), L-glutamine (2 mM), sodium bicarbonate (1 mM), glucose (4.5 g/L), HEPES (10 mM), and NaHCO_3_ (1.5 g/L). The cells were incubated at 37°C in a CO2 incubator. Cell confluence was determined using the trypan blue exclusion method. When the cell confluence reached 80-95%, the cells were sub-cultured. The culture medium (DMEM) was discarded, and the cells were washed with phosphate buffered saline (PBS) buffer. Trypsin solution was added to the culture flask and incubated at 37°C in the CO_2_ incubator for three to five minutes to detach the cells. After incubation, the cells were observed under a microscope to ensure detachment. To initiate subculturing, 5 mL of culture medium (DMEM) supplemented with 10% heat-inactivated FBS was added using a serological pipette. A549 cancer cells were seeded in 96-well flat-bottom microtiter plates at a density of approximately 10^6^ cells per well. The plates were incubated in a CO_2_ incubator for two days.

The cytotoxicity of synthesized AuNPs from the watery root extract of *Curcuma longa* and turmeric gold nanocapsules was assessed against Vero cells and malignant A549 cells using the tetrazolium (MTT) assay [22]. The cells were freshly cultured in 96-well flat-bottom plates and incubated overnight. The culture medium was then replaced with different concentrations of synthesized gold nanoparticles or turmeric gold nanocapsules and incubated overnight in a CO_2_ incubator. After overnight incubation, the cells were washed with phosphate buffer solution. Next, 20 µg/mL of tetrazolium dye was added to each well, and the plates were incubated for four hours in the dark. At the end of the incubation period, dimethyl sulfoxide (100 µL) was added to replace the tetrazolium dye, and the absorbance was measured at 595 nm using an enzyme-linked immunosorbent assay (ELISA) reader. The analysis of morphological changes and determination of the half maximal inhibitory concentration (IC50) value, which represents the concentration inhibiting half of viable cell development, was conducted to assess the cytotoxicity. A total of six well-flat-bottom cultured plates were used for the cultivation of A549 cancer cells (5 × 10^4^) along with turmeric gold nanocapsules. The cells were incubated overnight in a CO_2_ incubator. After the incubation, the cells treated with turmeric gold nanocapsules were washed for 10 minutes using an acetic acid and methanol solution. Subsequently, a counter stain of propidium iodide (50 μg/mL) was added to the culture plates, and the cells were incubated for 20 minutes. Following the incubation, the cells were observed under a fluorescence microscope. The staining procedure using acridine orange (AO) and ethidium bromide (EB) was employed to identify and evaluate the concentration of apoptosis and necrosis under a fluorescence microscope [23]. Six well flat-bottom plates were used for culturing the cancer cells, and they were incubated with turmeric gold nanocapsules overnight in a CO2 incubator. After the overnight incubation, the cells treated with turmeric gold nanocapsules were washed with phosphate buffer solution. Then, 5 μL of acridine orange was added as the primary fluorescent stain, followed by the addition of 5 μL of ethidium bromide as the counter stain. The cells were observed under a fluorescent microscope.

Analysis of DNA fragmentation

The technique described by Bortner et al. was utilized to isolate DNA from lung cancer cells treated with turmeric gold nanocapsules, followed by separation through agarose gel electrophoresis [24]. The DMEM growth medium containing a flat-bottom culture plate supplemented with fetal bovine serum was used to culture the lung malignant cells, which were incubated overnight in a CO_2_ incubator at the optimal temperature of 37°C. After the overnight incubation, the malignant cells treated with turmeric gold nanoparticles were washed with phosphate buffer solution. Next, a lysis buffer solution was added to the plates and gently mixed, followed by incubation at 37°C for 30 minutes. After the incubation period, the suspension was transferred to a centrifuge tube and proteinase-K was added for protein digestion, followed by incubation at 50°C for one hour. Subsequently, the sample was centrifuged with a combination of phenol-chloroform and isoamyl alcohol solution. The supernatant was collected in another centrifuge tube and incubated with a mixture of ethanol and sodium acetate at a low temperature for 30 minutes. After the incubation, centrifugation was performed, the supernatant was discarded, and the pellet was collected and incubated for an additional 30 minutes. The isolated DNA sample was loaded onto an agarose gel and separated through electrophoresis for one hour at 90V. Finally, the gel was visualized using a gel doc system.

## Results

**Figure 1:**
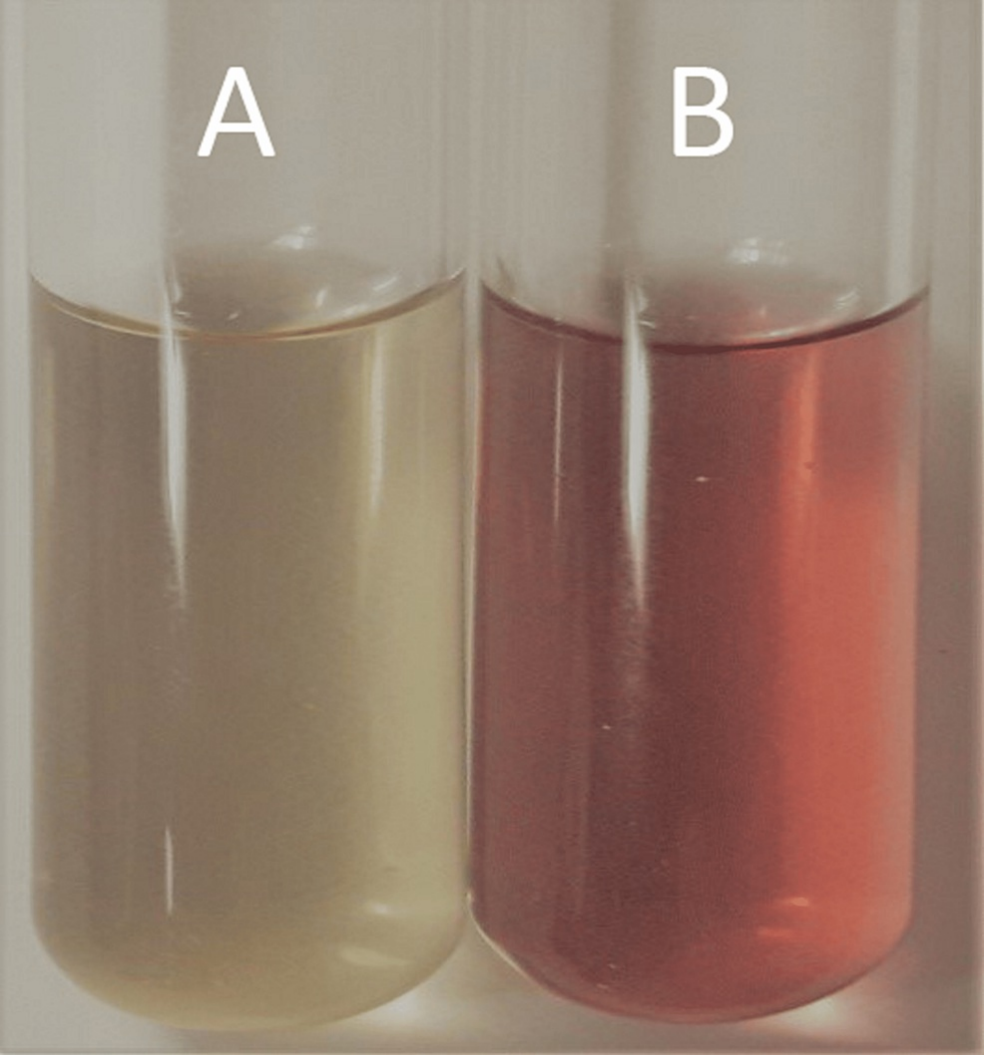
UV-Visible spectrum of Gold nanoparticles synthesized from Curcuma longa. A: Before synthesis of nanoparticles; B: After synthesis of nanoparticles

In our experiments, the color transformation of the gold nanoparticles from a light yellow aqueous solution to a ruby red shade within six hours demonstrated their ability to undergo a significant change in optical properties. This color change is often associated with the formation of gold nanoparticles and indicates the successful synthesis of nanoparticles in the solution (Figure 1). 

**Figure 2:**
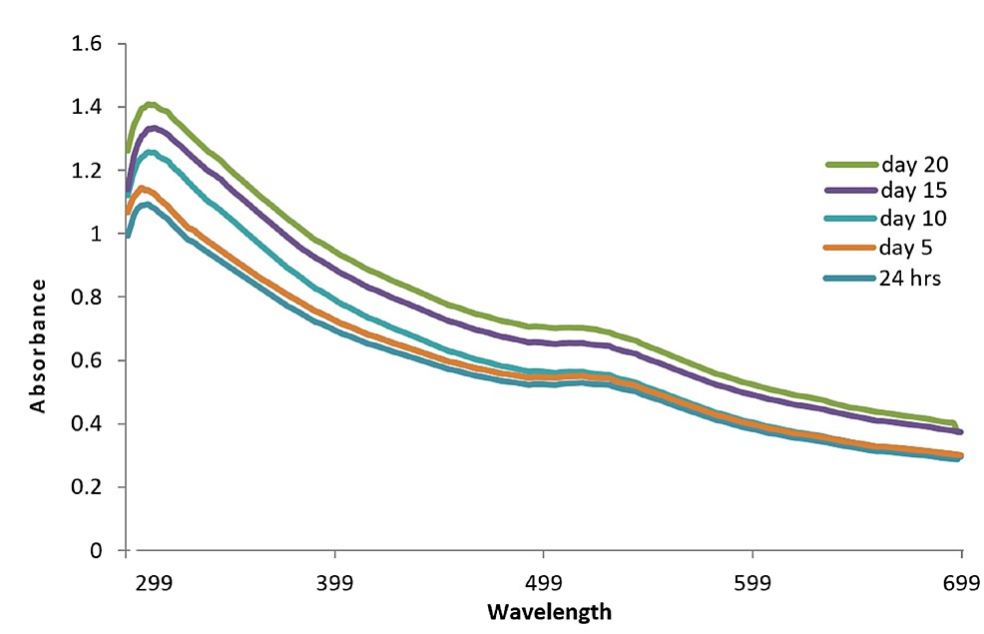
UV-Vis spectra of synthesized nanoparticles at different time intervals.

To optimize the synthesis process, different time intervals were tested to achieve the maximum yield of gold nanoparticles. The reaction was monitored at various time spans ranging from 24 hours to 25 days. UV-Vis spectral absorption pattern was observed to assess the stability and formation of the nanoparticles over time. Minor alterations in the absorption peaks at 520 nm, 522 nm, and 527 nm were observed post-48 hours which remained consistent for 25 days, indicating the robust stability of the biosynthesized gold nanoparticles. Transmission electron microscope (TEM) analysis was carried out to examine the morphology and size of the photosynthesized gold nanoparticles. The TEM images revealed that the gold nanoparticles exhibited a spherical shape, and their size was found to range between 25-35 nm (Figure 2).

**Figure 3:**
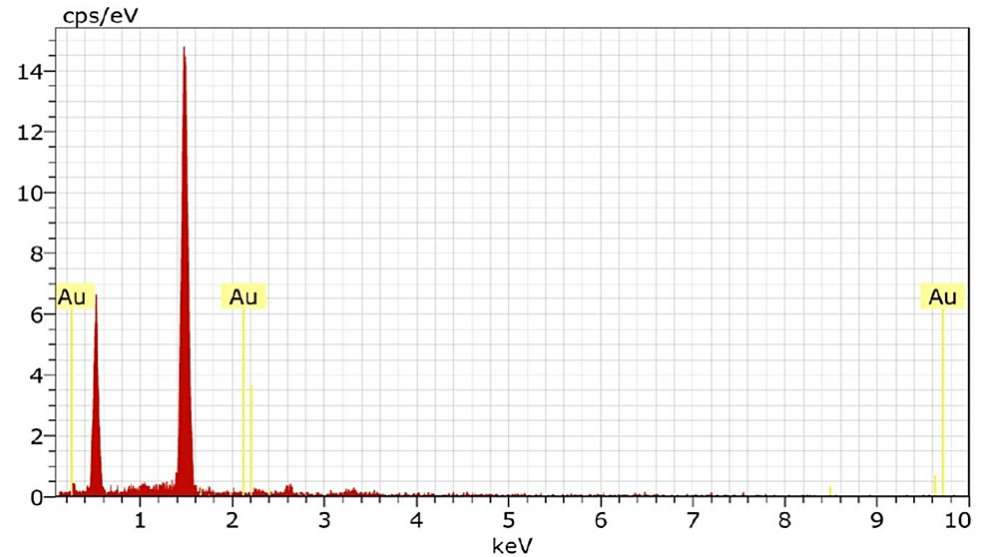
Energy dispersive X-ray analysis of phytosynthesized gold nanoparticles.

Energy dispersive x-ray analysis (EDX) was performed to determine the elemental composition of the synthesized gold nanoparticles. The EDX results confirmed that the integrated nanoparticles were free from impurities, as only a single signal corresponding to gold (Au) was detected. Additionally, the presence of oxygen (O) and carbon (C) peaks alongside the gold signal suggested the presence of phytoconstituents on the surface of the synthesized gold nanoparticles. This indicates that the nanoparticles were capped or surrounded by organic molecules, which may contribute to their stability and potential bioactivity (Figure 3).

**Figure 4:**
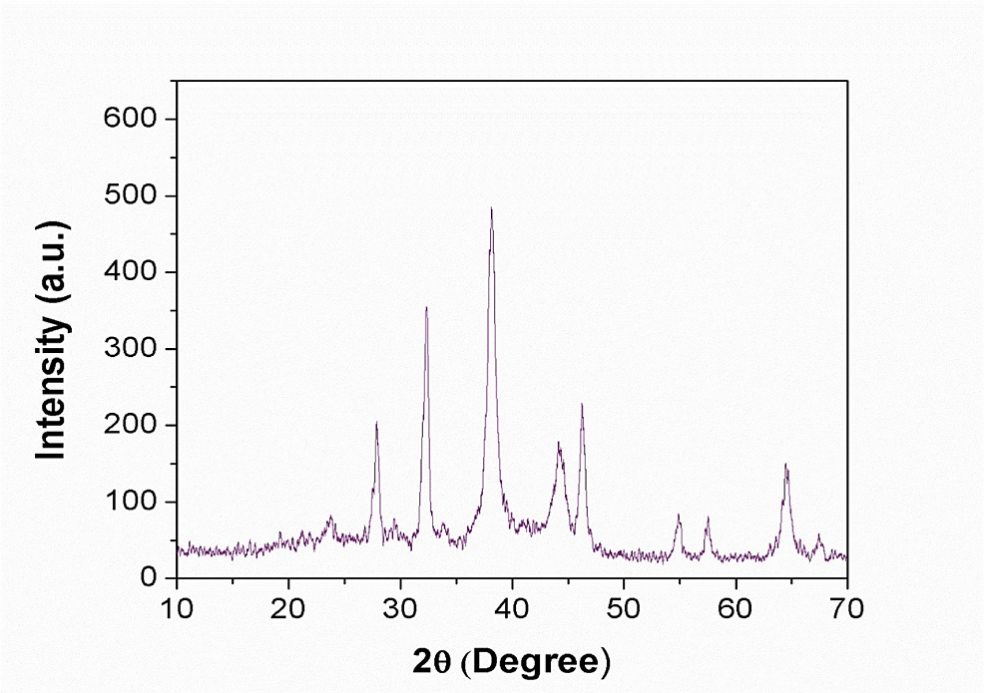
X-Ray Diffraction analysis phytosynthesized gold nanoparticles.

X-ray diffraction studies (XRD) were conducted to analyze the crystal structure of the gold nanoparticles. The XRD pattern exhibited characteristic peaks at 28.0°, 33.04°, 38.3°, and 48.2°, which corresponded to the (200), (350), (450), and (205) miller indices, respectively. The absence of additional peaks indicated that the synthesized gold nanoparticles were highly pure and did not contain any impurities. Furthermore, the average size of the gold nanoparticles was determined to be 11 nm, which falls within the desired nanoscale range (Figure 4).

**Figure 5:**
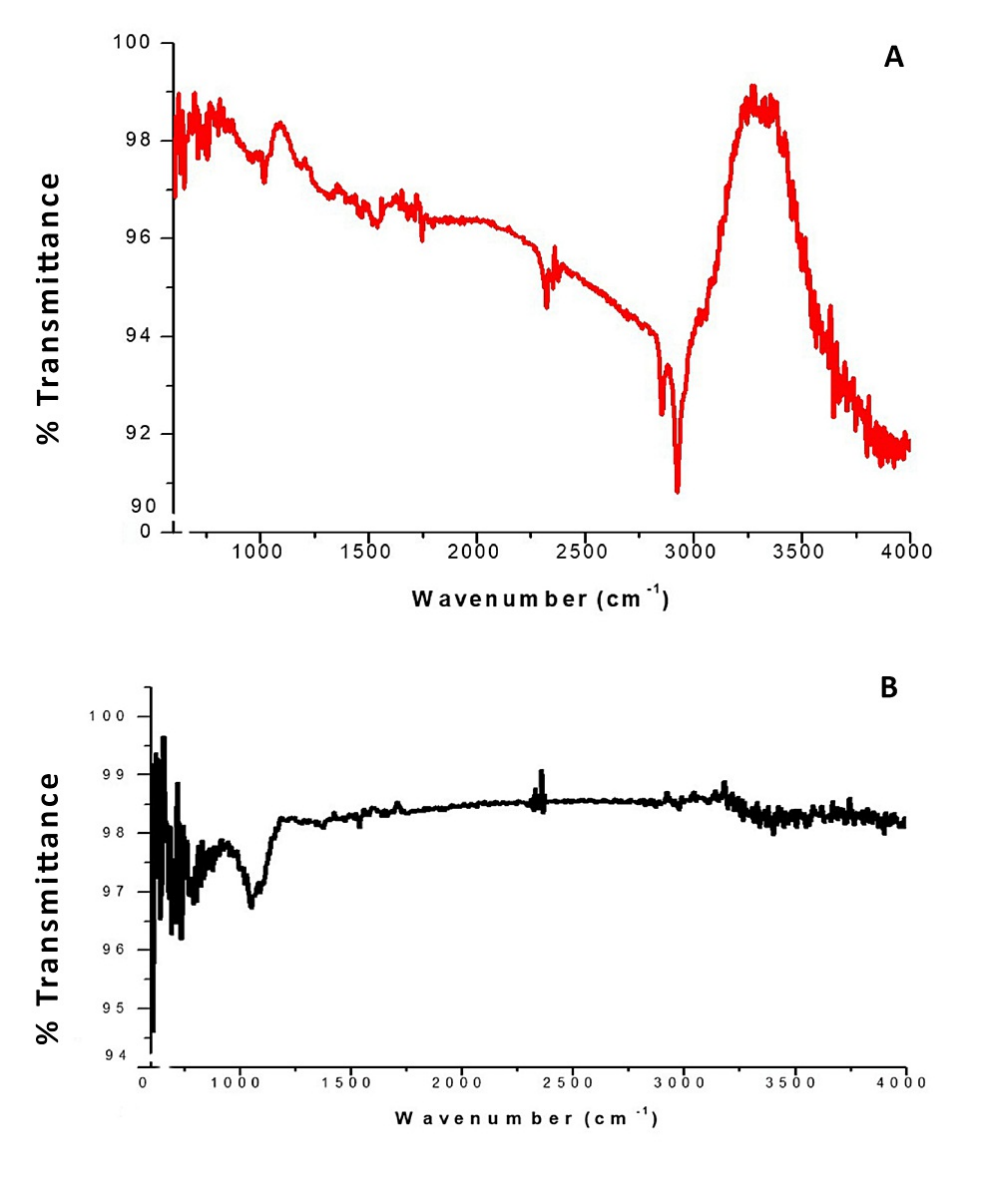
FTIR spectrum analysis The graph above in red represents the Fourier Transform Infrared Spectroscopy (FTIR) spectrum of synthesized Gold nanoparticles from Curcuma longa root aqueous extract. Whereas the graph below in black represents the FTIR spectrum of ethyl cellulose (EC) mediated gold/turmeric nanocapsules.

Fourier transform infra-red (FTIR) analysis was performed to investigate the functional groups responsible for the proper binding of the gold nanoparticles with turmeric powder. The FTIR spectra showed minimal changes in the coupling groups, suggesting that the binding interactions between the nanoparticles and turmeric powder remained relatively stable. Notably, a peak shift around 3200 cm^-1^ was observed, indicating the presence of carboxyl or alcoholic groups that were responsible for the effective binding of gold nanoparticles with turmeric powder, facilitating the formation of gold nanocapsules (Figure 5).

The DPPH free radical scavenging assay was conducted to evaluate the antioxidant activity of the synthesized gold nanoparticles. The results demonstrated that the incorporated gold nanoparticles exhibited concentration-dependent radical scavenging activity. This indicates that the synthesized nanoparticles possess potential antioxidant properties, which can be attributed to the presence of phytoconstituents from *Curcuma longa* extract (Table 1). 

**Table 1:** DPPH free radical scavenging assay for anti-oxidant activity. DPPH: 1,1-diphenyl-2-picrylhydrazyl; GNPs- gold nanoparticles

Test tube	DPPH(ml)	GNPs	Incubation Time (min)	Absorption	% Inhibition
Control (1)	2	0	30	0.861	0%
2	2	25	30	0.770	10.50%
3	2	50	30	0.710	17.50%
4	2	100	30	0.510	40.70%
5	2	250	30	0.345	59.90%
6	2	500	30	0.200	76.80%

**Figure 6:**
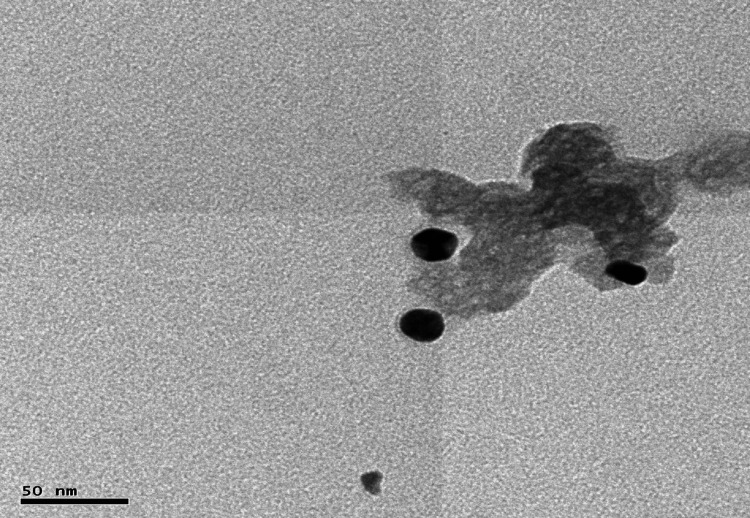
Transmission electron microscopy (TEM) Analysis of gold nanocapsules.

TEM analysis of the gold nanocapsules confirmed their spherical shape and revealed a diameter ranging from 10-20 nm. The images also showed that the polymer-based nanocapsules contained dispersed metal nanoparticles. This suggests that the encapsulation process successfully incorporated the gold nanoparticles within the polymer matrix, resulting in the formation of stable gold nanocapsules (Figure 6).

**Figure 7:**
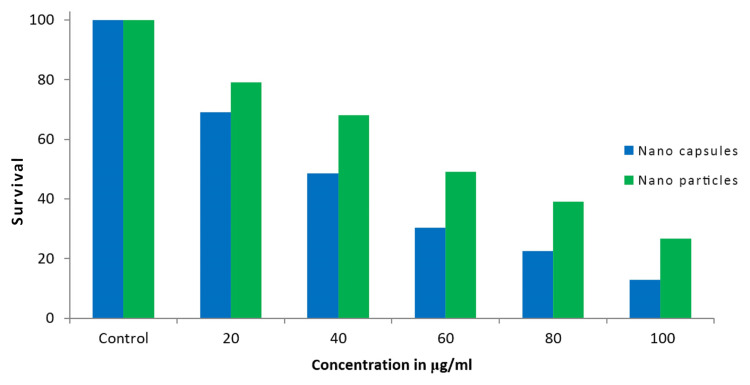
Cell survival analysis using MTT assay MTT Assay showing the effect of different concentrations of gold nanoparticles and gold nanocapsules on A549 cell lines at 24hrs. MTT: 3-(4,5-dimethylthiazol-2-yl)-2,5-diphenyltetrazolium bromide.

Cytotoxicity studies were performed on the human lung cancer cell line A549 using both the gold nanoparticles and gold nanocapsules synthesized with turmeric. The results indicated that the gold nanocapsules exhibited stronger cytotoxic effects compared to the gold nanoparticles. The IC50 values for the gold nanocapsules and gold nanoparticles were recorded at 40μg/ml and 60μg/ml, respectively. These concentrations resulted in a significant decrease in cell viability compared to the control group, indicating the potential of the synthesized nanoparticles for cancer therapy applications (Figure 7).

**Figure 8:**
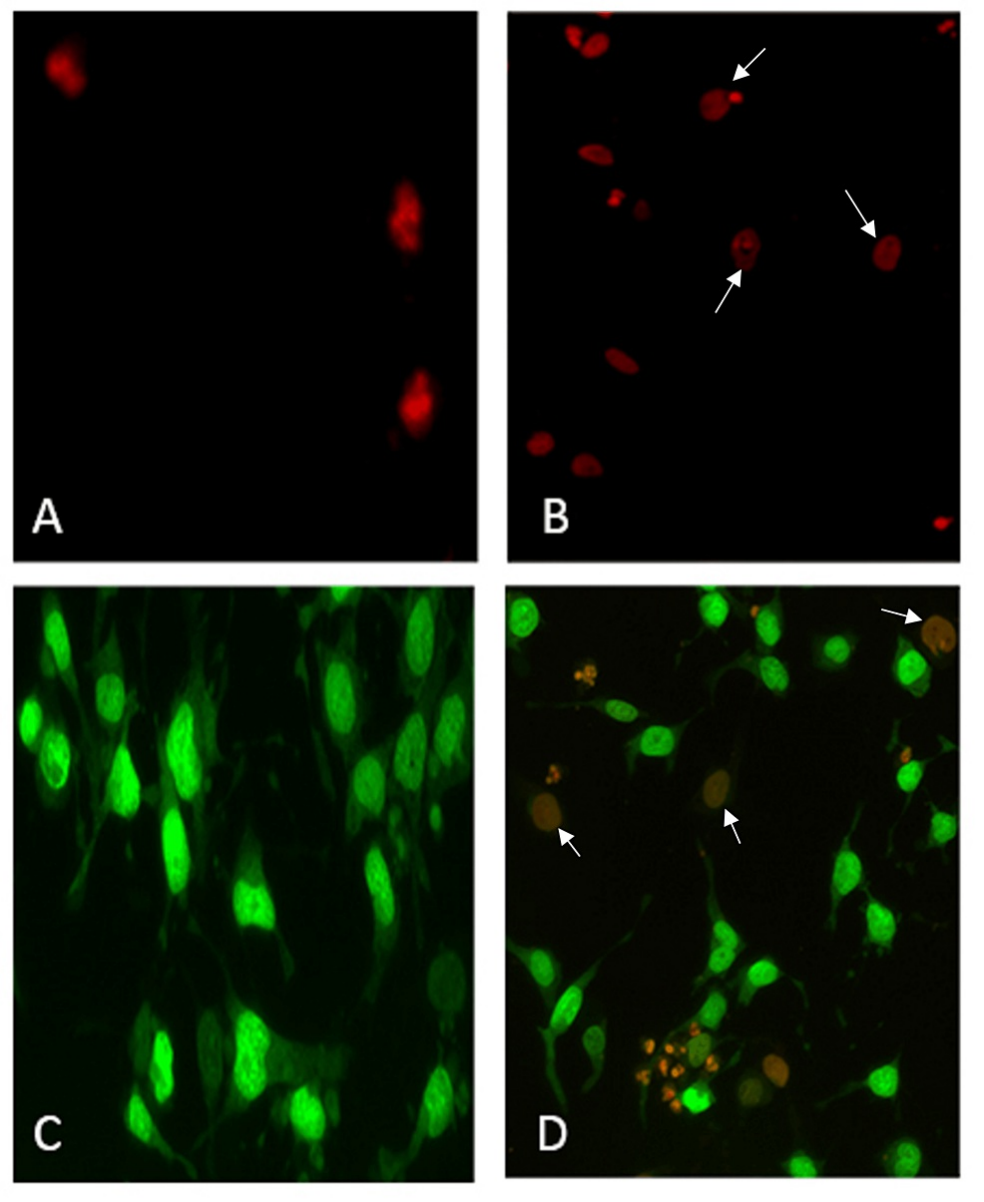
Cellular morphological changes for apoptotic analysis by fluorescence microscopy A  - A549 control cells stained with propidium iodide; B - effect of gold nanocapsules at 50 ug/ml on A549 cells at 24 h with propidium iodide stain; C- A549 control cells stained with acridine orange (AO) and ethidium bromide (EB); D - effect of gold nanocapsules at 60ug/ml on nuclei of A549 cells at 24 hrs stained with acridine orange (AO) and ethidium bromide (EB). Arrowheads indicate positively stained nuclei in figures B and D.

Fluorescence microscopy analysis using propidium iodide dye revealed an increase in propidium iodide-positive cells in the gold nanocapsule-treated A549 cells, indicating apoptotic changes and potential mitochondrial damage. This suggests that the gold nanocapsules induce apoptosis in the cancer cells, leading to cell death. Further morphological analysis of A549 cells using acridine orange and ethidium bromide staining provided additional evidence of apoptotic changes in the gold nanocapsule-treated cells compared to the control group. The gold nanocapsule-treated cells displayed distinct morphological features characteristic of apoptosis, such as nuclear condensation and fragmentation (Figure 8).

**Figure 9:**
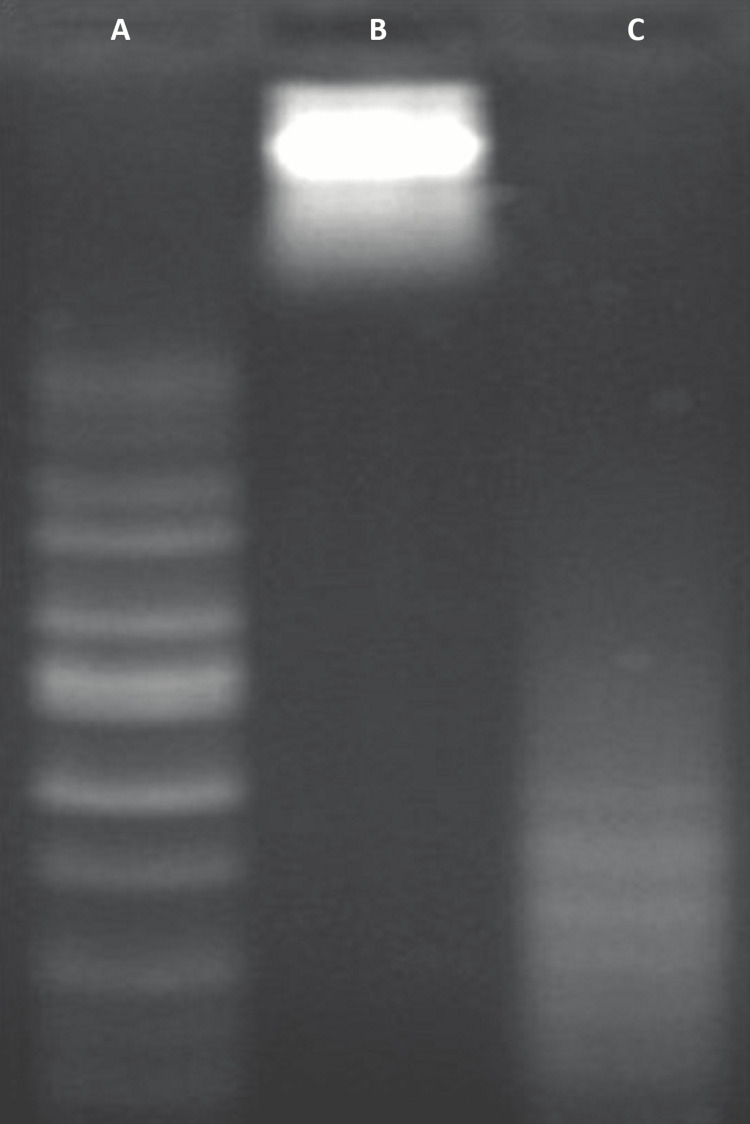
DNA Fragmentation Analysis A - 1kb DNA ladder (wide range from 250bp (base pairs) to 10,000bp serving as control; B - total genomic DNA from untreated A549 cells; C - total genomic DNA from treated A549 cells.

DNA fragmentation analysis further supported the occurrence of apoptosis in the gold nanocapsule-treated A549 cells. The presence of fragmented DNA in the treated cells indicated the activation of apoptotic pathways and the potential efficacy of the gold nanocapsules in inducing programmed cell death (Figure 9).

## Discussion

The synthesis and characterization of gold nanoparticles (AuNPs) and gold nanocapsules using *Curcuma longa* extract have been successfully carried out in this study. The UV-Vis spectroscopy results confirmed the formation of AuNPs, as evidenced by the absorption peak at 520-530 nm, which corresponds to the surface plasmon resonance of gold nanoparticles (Mie's hypothesis) [25]. The color change of the gold nanoparticles from light yellow to ruby red within six hours indicates the successful synthesis and stability of the nanoparticles. The absorbance of *Curcuma longa* watery solution with AuNPs was observed under UV-Vis spectrum, showing absorption at 530 nm after six hours of incubation. This suggests that the nanoparticles have strong absorption properties within the visible range. To optimize the synthesis process, various time intervals were tested, and the results showed that the maximum amalgamation of AuNPs was achieved within 48 hours of incubation. UV-Vis analysis revealed slight modifications in the absorption peaks at 520 nm, 522 nm, and 527 nm, indicating the stability of the biosynthesized gold nanoparticles. The absence of a peak at 0 minutes suggests that the nanoparticles require a certain incubation time for their formation and stabilization.

TEM analysis was performed to analyze the morphology and size of the synthesized AuNPs. The images confirmed that the AuNPs were spherically shaped, with sizes ranging from 25-35 nm. This is consistent with previous studies reporting the size range of synthesized gold nanoparticles [26]. The spherical shape and uniform size distribution are favorable characteristics for the potential applications of these nanoparticles. EDX analysis confirmed the purity of the synthesized AuNPs, as only a single signal corresponding to gold was detected. The presence of oxygen and carbon peaks alongside the gold signal suggests that the nanoparticles were capped by phytoconstituents, which may contribute to their stability and bioactivity. XRD analysis confirmed the crystalline structure of the gold nanoparticles, with peaks observed at specific miller indices. The absence of additional peaks indicates the high purity of the synthesized nanoparticles. The average size of the nanoparticles, determined through XRD analysis using the Scherrer equation, was found to be 11 nm [27]. The absence of impurities further supports the suitability of these nanoparticles for various applications. FTIR analysis was performed to investigate the functional groups involved in the binding of gold nanoparticles with turmeric powder and the formation of gold nanocapsules. The observed peak shifts in the spectra suggested the involvement of carboxyl or alcoholic groups in the proper binding of the nanoparticles, leading to the formation of stable nanocapsules. This finding is in line with previous studies that have reported the successful synthesis of gold nanocapsules using turmeric as a bioactive compound [28]. The DPPH free radical scavenging assay demonstrated the antioxidant activity of the synthesized gold nanoparticles. The concentration-dependent reduction of the DPPH radical indicates their potential as effective antioxidants. This finding aligns with previous studies highlighting the antioxidant properties of gold nanoparticles [29].

The cytotoxicity of the synthesized AuNPs and gold nanocapsules was evaluated against A549 lung cancer cells. The MTT assay results showed concentration-dependent cytotoxic effects for both nanoformulations. The IC50 values were determined to be 60 μg/ml for AuNPs and 40 μg/ml for gold nanocapsules, indicating a higher potency of the nanocapsules in inhibiting the viability of A549 cells. These results are consistent with previous studies that have reported the cytotoxic effects of gold nanoparticles on cancer cells [30]. The enhanced cytotoxicity of the nanocapsules could be attributed to the sustained release of bioactive compounds from turmeric, the size and surface properties of the nanoparticles, and their interactions with the cancer cells. Fluorescence microscopy analysis using propidium iodide staining revealed an increase in the number of positively stained cells in the gold nanocapsule-treated A549 cells, indicating apoptotic changes and potential mitochondrial damage. The acridine orange/ethidium bromide dual staining assay further supported the induction of apoptosis in the treated cells, as evidenced by distinct morphological features associated with apoptosis. The DNA fragmentation analysis provided additional evidence of apoptotic cell death in the treated cells, as fragmented DNA is a characteristic hallmark of apoptosis [24]. These findings collectively demonstrate the successful synthesis of stable gold nanoparticles and gold nanocapsules using Curcuma longa extract. The nanoparticles exhibited strong absorption properties, good stability, and cytotoxic effects against A549 lung cancer cells. The incorporation of turmeric into the nanocapsules enhanced their anticancer activity, potentially through the sustained release of bioactive compounds. These results align with previous studies highlighting the potential of gold nanoparticles and turmeric in cancer therapy. Further studies are warranted to explore the underlying mechanisms of action and evaluate the therapeutic potential of these nanoformulations in vivo.

## Conclusions

In conclusion, the study successfully synthesized gold nanoparticles and gold nanocapsules using *Curcuma longa* extract. The combination of turmeric gold nanocapsules exhibited more potent anticancer activity on the lung cancer cell line compared to biologically synthesized gold nanoparticles. These findings highlight the enhanced therapeutic efficacy of incorporating turmeric into the nanocapsules. The nanoparticles demonstrated strong absorption properties, antioxidant activity, and concentration-dependent cytotoxic effects on A549 lung cancer cells. The induction of apoptosis further supports their potential for cancer therapy. Future research should focus on elucidating the underlying mechanisms and optimizing the formulation of gold/turmeric nanocapsules to improve anticancer treatments.

## References

[1] Barta JA, Powell CA, Wisnivesky JP (2019). Global epidemiology of lung cancer. Ann Glob Health.

[2] Torricelli P, Antonelli F, Ferorelli P, Borromeo I, Shevchenko A, Lenzi S, De Martino A (2020). Oral nutritional supplement prevents weight loss and reduces side effects in patients in advanced lung cancer chemotherapy. Amino Acids.

[3] Monteiro Lde S, Bastos KX, Barbosa-Filho JM, de Athayde-Filho PF, Diniz Mde F, Sobral MV (2014). Medicinal plants and other living organisms with antitumor potential against lung cancer. Evid Based Complement Alternat Med.

[4] Doroudian M, O' Neill A, Mac Loughlin R, Prina-Mello A, Volkov Y, Donnelly SC (2021). Nanotechnology in pulmonary medicine. Curr Opin Pharmacol.

[5] Ibáñez MD, Blázquez MA (2020). Curcuma longa l. Rhizome essential oil from extraction to its agri-food applications. A review. Plants (Basel).

[6] Aggarwal BB, Yuan W, Li S, Gupta SC (2013). Curcumin-free turmeric exhibits anti-inflammatory and anticancer activities: Identification of novel components of turmeric. Mol Nutr Food Res.

[7] Song W, Su X, Gregory DA, Li W, Cai Z, Zhao X (2018). Magnetic alginate/chitosan nanoparticles for targeted delivery of curcumin into human breast cancer cells. Nanomaterials (Basel).

[8] Moballegh Nasery M, Abadi B, Poormoghadam D (2020). Curcumin delivery mediated by bio-based nanoparticles: a review. Molecules.

[9] Yavarpour-Bali H, Ghasemi-Kasman M, Pirzadeh M (2019). Curcumin-loaded nanoparticles: a novel therapeutic strategy in treatment of central nervous system disorders. Int J Nanomedicine.

[10] Chen Y, Lu Y, Lee RJ, Xiang G (2020). Nano encapsulated curcumin: and its potential for biomedical applications. Int J Nanomedicine.

[11] Foo YY, Periasamy V, Kiew LV, Kumar GG, Malek SN (2017). Curcuma mangga-mediated synthesis of gold nanoparticles: characterization, stability, cytotoxicity, and blood compatibility. Nanomaterials (Basel).

[12] Markman JL, Rekechenetskiy A, Holler E, Ljubimova JY (2013). Nanomedicine therapeutic approaches to overcome cancer drug resistance. Adv Drug Deliv Rev.

[13] Mendes BB, Sousa DP, Conniot J, Conde J (2021). Nanomedicine-based strategies to target and modulate the tumor microenvironment. Trends Cancer.

[14] Boisselier E, Astruc D (2009). Gold nanoparticles in nanomedicine: preparations, imaging, diagnostics, therapies and toxicity. Chem Soc Rev.

[15] Sardar R, Funston AM, Mulvaney P, Murray RW (2009). Gold nanoparticles: past, present, and future. Langmuir.

[16] Erdoğar N, Akkın S, Bilensoy E (2018). Nanocapsules for drug delivery: an updated review of the last decade. Recent Pat Drug Deliv Formul.

[17] Lima AL, Gratieri T, Cunha-Filho M, Gelfuso GM (2022). Polymeric nanocapsules: a review on design and production methods for pharmaceutical purpose. Methods.

[18] Mahnaj T, Ahmed SU, Plakogiannis FM (2013). Characterization of ethyl cellulose polymer. Pharm Dev Technol.

[19] Cui Y, Zhang H, Wang J (2022). Preparation of ethyl cellulose particles with different morphologies through microfluidics. Soft Matter.

[20] Deng S, Gigliobianco MR, Censi R, Di Martino P (2020). Polymeric nanocapsules as nanotechnological alternative for drug delivery system: current status, challenges and opportunities. Nanomaterials (Basel).

[21] Kedare SB, Singh RP (2011). Genesis and development of DPPH method of antioxidant assay. J Food Sci Technol.

[22] Kumar P, Nagarajan A, Uchil PD (2018). Analysis of cell viability by the MTT assay. Cold Spring Harb Protoc.

[23] Bortner CD, Oldenburg NB, Cidlowski JA (1995). The role of DNA fragmentation in apoptosis. ScienceDirect.

[24] Kasibhatla S, Amarante-Mendes GP, Finucane D, Brunner T, Bossy-Wetzel E, Green DR (2006). Acridine orange/Ethidium bromide (AO/EB) staining to detect apoptosis. CSH Protoc.

[25] Oliveira AEF, Pereira AC, Resende MAC, Ferreira LF. Gold Nanoparticles (2023). A didactic step-by-step of the synthesis using the turkevich method, mechanisms, and characterizations. Analytica.

[26] Young NP, van Huis MA, Zandbergen HW, Xu H, Kirkland AI (2010). Transformations of gold nanoparticles investigated using variable temperature high-resolution transmission electron microscopy. Ultramicroscopy.

[27] Muniz FT, Miranda MA, Morilla Dos Santos C, Sasaki JM (2016). The Scherrer equation and the dynamical theory of x-ray diffraction. Acta Crystallogr A Found Adv.

[28] Hettiarachchi SS, Dunuweera SP, Dunuweera AN, Rajapakse RM (2021). Synthesis of curcumin nanoparticles from raw turmeric rhizome. ACS Omega.

[29] Rajeshkumar S, Parameswari RP, Jayapriya J (2022). Apoptotic and antioxidant activity of gold nanoparticles synthesized using marine brown seaweed: an in vitro study. Biomed Res Int.

[30] Majoumouo MS, Sharma JR, Sibuyi NR, Tincho MB, Boyom FF, Meyer M (2020). Synthesis of biogenic gold nanoparticles from terminalia mantaly extracts and the evaluation of their in vitro cytotoxic effects in cancer cells. Molecules.

